# The Soybean GmNAC019 Transcription Factor Mediates Drought Tolerance in *Arabidopsis* in an Abscisic Acid-Dependent Manner

**DOI:** 10.3390/ijms21010286

**Published:** 2019-12-31

**Authors:** Xuan Lan Thi Hoang, Nguyen Cao Nguyen, Yen-Nhi Hoang Nguyen, Yasuko Watanabe, Lam-Son Phan Tran, Nguyen Phuong Thao

**Affiliations:** 1 Applied Biotechnology for Crop Development Research Unit, School of Biotechnology, International University–Vietnam National University HCMC, Ho Chi Minh 700000, Vietnam; htlxuan@hcmiu.edu.vn (X.L.T.H.); nguyencaonguyenbiotech@gmail.com (N.C.N.); nhynhi.biotech@gmail.com (Y.-N.H.N.); 2 Stress Adaptation Research Unit, RIKEN Center for Sustainable Resource Science, 1-7-22, Suehiro-cho, Tsurumi, Yokohama 230-0045, Japan; yasuko.watanabe@riken.jp; 3 Institute of Research and Development, Duy Tan University, 03 Quang Trung, Da Nang 550000, Vietnam

**Keywords:** ABA-mediated response, drought tolerance, *GmNAC019*, transgenic *Arabidopsis*

## Abstract

Being master regulators of gene expression, transcription factors (TFs) play important roles in determining plant growth, development and reproduction. To date, many TFs have been shown to positively mediate plant responses to environmental stresses. In the current study, the biological functions of a stress-responsive NAC [NAM (No Apical Meristem), ATAF1/2 (*Arabidopsis* Transcription Activation Factor1/2), CUC2 (Cup-shaped Cotyledon2)]-TF encoding gene isolated from soybean (*GmNAC019*) in relation to plant drought tolerance and abscisic acid (ABA) responses were investigated. By using a heterologous transgenic system, we revealed that transgenic *Arabidopsis* plants constitutively expressing the *GmNAC019* gene exhibited higher survival rates in a soil-drying assay, which was associated with lower water loss rate in detached leaves, lower cellular hydrogen peroxide content and stronger antioxidant defense under water-stressed conditions. Additionally, the exogenous treatment of transgenic plants with ABA showed their hypersensitivity to this phytohormone, exhibiting lower rates of seed germination and green cotyledons. Taken together, these findings demonstrated that GmNAC019 functions as a positive regulator of ABA-mediated plant response to drought, and thus, it has potential utility for improving plant tolerance through molecular biotechnology.

## 1. Introduction

Throughout their lives, plants’ growth and development are always governed by living conditions. Like animals, plants also possess distinct innate responsive mechanisms to minimize the negative impacts of their environments [1]. The responses of plants to environmental stresses are the outcomes of their capability of sensing and subsequently transducing the stress information toward the plant nucleus to regulate the genetic machinery [2,3]. Currently, it is known that the plant defense deploys a sophisticated network consisting of diverse proteins and metabolites [4]. Among the identified participants, transcription factors (TFs) play a vital role in supporting plant responses to environmental stresses, apparently due to their ability of binding to gene promoter regions and interacting with other proteins to regulate gene expression and protein functions [2,5,6]. Examples of several TF families found in plants which are important in both biotic and abiotic stress responses are AP2/ERF (APETALA2/ethylene-responsive element binding factor) [7,8], NAC (NAM, ATAF1/2, CUC2) [9,10], bZIP (basic leucine zipper) [11,12], MYB (myeloblastosis) [13,14], WRKY (tryptophan-arginine-lysine-tyrosine) [15,16] and zinc-finger proteins [17,18,19].

With respect to the NAC TF family, functional characterization studies revealed that the NAC members participate in monitoring a wide spectrum of biological processes. Specifically, they have been shown to regulate embryo development [20,21], root development [22,23], shoot development [24], secondary cell wall formation [25,26], reproductive organ development [27,28], fiber development (e.g., in cotton) [29], trichome development [30,31], ripening [32] and senescence [33]. Their regulatory mechanisms have also been demonstrated to be coordinated by various hormone signaling pathways, including abscisic acid (ABA)- [34], jasmonic acid- [35,36], auxin- [37,38], ethylene- [39,40] and gibberellic acid-dependent [41] signaling pathways. Besides, transcriptome profiling studies revealed that many NAC members are responsive to environmental stimuli, including drought [42,43]. Structural analyses of typical NAC proteins indicated that the domain interacting with DNA sequences (*cis*-elements) is located in the N-terminal region, and is composed of several motifs, while the regulatory domain can be found in the C-terminal region [2,5]. Detailed processes of DNA binding and the protein interactions of many NAC TFs have been studied and reported in many published works [44,45,46,47]. For example, the interactions between the ZFHD1 (zinc finger homeodomain 1) and ANAC019, ANAC055 or ANAC072, were studied using yeast-two hybrid and transactivation assays in *Arabidopsis* protoplasts, and their interactions were shown to have essential roles in improving drought tolerance of *Arabidopsis* plants [48].

A great number of reports have shown that enhancement of plant resistance to drought could be achieved by manipulating certain *NAC* genes. In *Arabidopsis*, overexpression of the *Arabidopsis* drought-inducible genes *ANAC019*, *ANAC055* or *ANAC072* could enhance drought tolerance in the transgenic plants [49]. Transforming *Arabidopsis* plants using *NAC*s from other crop species, such as wheat (*Triticum aesitivum*) *TaNAC29* [50] and maize (*Zea mays*) *ZmSNAC1* [51], also showed better tolerance of transgenic plants to drought compared with non-transgenic plants. Similar successes have also been obtained in other plant systems. In rice (*Oryza sativa*), the NAC TFs functioning as positive mediators in plant response to drought include SNAC1 [52], ONAC022 [53], OsNAC6 [54] and SNAC3 [55]. Likewise, in transgenic tobacco, ectopic expression of peanut (*Arachis hypogaea*) *AhNAC3* [56] or wheat *TaNAC2a* [57] could promote drought tolerance in the constructed plants. Functional characterizations of the examined NAC TFs indicated that they participate in regulating diverse plant defending and adaptive activities, which are related to reactive oxygen species (ROS) removal, germination, root development, transpiration, photosynthetic performance, senescence, membrane transport and stabilization, nutrient remobilization and production of carbohydrate and osmo-protective compounds [51,54,58,59,60,61,62,63]. Furthermore, the NAC-induced resistance coupled with minimized yield loss in transgenic plants upon stress exposure has also been reported in several studies [62,64,65]. These lines of evidence highlight the NAC TF family as an important resource of candidate genes that can be used for improvement of crop yields under water scarcity by genetic engineering.

In soybeans (*Glycine max*), a number of *GmNAC* genes have been identified to be drought and/or dehydration-responsive [43,66,67,68]. Among these, the *GmNAC019*/*Glyma04g38990.1* [43] (the so-called *GmNAC020* in [5]) was reported as one of the *NAC* genes induced by dehydration. In addition, GmNAC019 possesses transactivation, as evidenced by a yeast one-hybrid assay [5]. The latest investigations using two soybean cultivars with contrasting drought-tolerant phenotypes revealed positive correlation of *GmNAC019* expression and the drought tolerance capacity, suggesting its beneficial regulatory functions for drought tolerance [67,68]. With great interest in identifying the roles of GmNAC019 in regulating plant drought responses, in the present study, we conducted a detailed functional analysis of GmNAC019 by using transgenic *Arabidopsis* plants ectopically expressing *GmNAC019*.

## 2. Results and Discussion

### 2.1. Transgenic Arabidopsis Plants Ectopically Expressing GmNAC019 Exhibited a Smaller Phenotype

**Figure 1:**
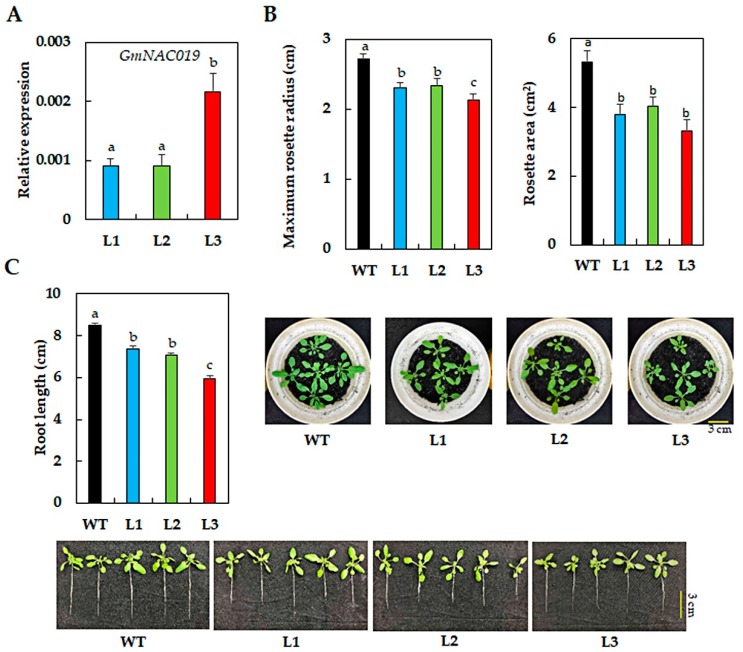
Phenotypes of three transgenic *Arabidopsis* lines (L1, L2 and L3) carrying *35S:GmNAC019* under normal conditions. (**A**) Relative expression of *GmNAC019* detected in the transgenic lines but not wild-type (WT) plants using rosette leaves (*n* = 3). (**B**) Maximum rosette radius and average rosette area (*n* = 10) with illustrated photographs. (**C**) Average tap root length (*n* = 10). Four-week-old plants were used in the examination. Error bars represent SEs. Different letters indicate significant differences (*p*-value < 0.05) among the genotypes, as analyzed by a Duncan’s multiple range test.

To understand the regulatory functions of the drought-inducible *GmNAC019* gene in mediating plant response to drought, we constructed transgenic *Arabidopsis* plants ectopically expressing *GmNAC019* using the *CaMV 35S* promoter. Through screening for the homozygous transgenic plants that carried a single copy of transgene, three independent lines were obtained and designated L1, L2 and L3. The expression of the transgene was detected in these three lines, but not in the wild-type (WT) plants (Figure 1A). According to the results, L3 had the *GmNAC019* expression approximately 2.4-fold higher than the other two transgenic lines. In comparison with L1 and L2, L3 displayed a significantly smaller rosette (Figure 1B) and shorter root length (Figure 1C). When compared with WT plants, all three transgenic lines were smaller in size, as indicated by examination of several phenotypic parameters, including rosette radius and area, and root length (Figure 1B,C). These findings imply that constitutive expression of *NAC* genes using the *CaMV 35S* promoter might result in growth retardation in homologous/heterologous transgenic systems, as also observed in many previously published reports, such as ectopic expression of *GmNAC085* [69] or *GmNAC109* [70] in *Arabidopsis*, and overexpression of *Thellungiella halophila TsNAC1* in *T. halophila* [71] or *ANAC036* in *Arabidopsis* [72]. Additional analyses from some of these studies reveal that the restriction in cell size and expansion was a cause of growth retardation, probably due to the strong activity of this promoter [71,72]. Thus, other constitutive promoters with lower activities than that of *CaMV 35S* might be considered for ectopic expression/overexpression of *NAC* genes, and perhaps TF-encoding genes belonging to other TF families, to avoid any growth penalty effects on the transgenic plants.

### 2.2. GmNAC019-Transgenic Plants Displayed Better Drought Tolerance Potential

**Figure 2:**
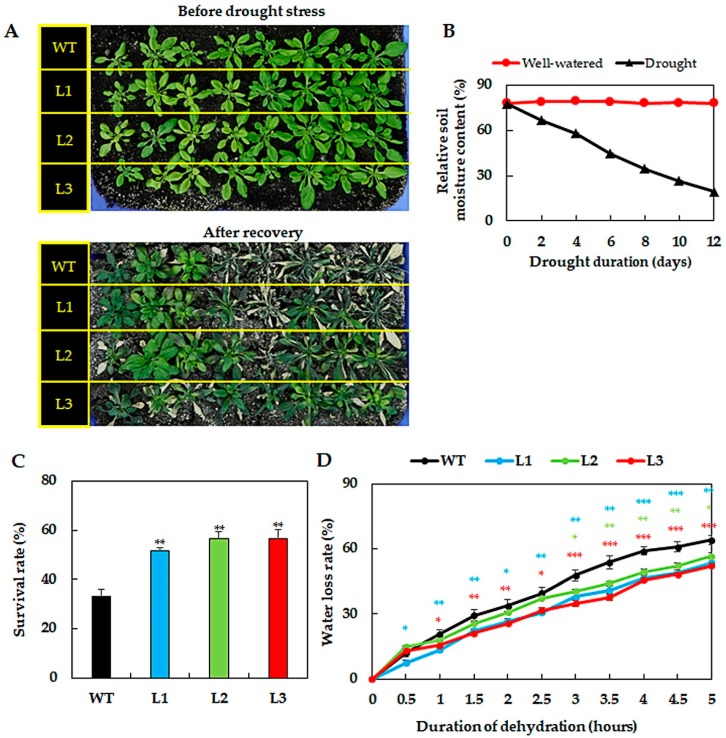
Drought tolerance evaluation of *GmNAC019*-transgenic *Arabidopsis* plants based on drought survival rate and water loss rate. (**A**) Photographs of wild-type (WT) and three independent transgenic lines (L1, L2 and L3) before drought treatment and after 12-day soil-drying followed by a 3-day re-watering application. Four-week-old plants were subjected to drought treatment. (**B**) Average relative soil moisture contents that were measured over the course of drought treatment (*n* = 6). (**C**) Plant survival percentages after recovery (*n* = 3). (**D**) Average water loss rates in leaves excised from four-week-old plants over the course of 5-h dehydration (*n* = 9). Error bars represent SEs. Asterisks indicate significant differences (* *p*-value < 0.05; ** *p*-value < 0.01; *** *p*-value < 0.001) between each transgenic line and the WT counterpart at the same treatment conditions, according to a Student’s *t*-test.

To determine whether GmNAC019 plays a role in drought tolerance, we evaluated the survival capacities of the *GmNAC019*-transgenic L1, L2 and L3 plants under water deficit conditions by performing a drought tolerance assay using the soil-drying method. After 12 days of drought exposure, markedly enhanced survival rates were observed in all three transgenic lines compared with that of WT plants (Figure 2A–C). According to our data, more than 50% of the transgenic plants but only around 30% of the WT plants were able to recover after re-watering (Figure 2C). Further examination of the water loss rates in the leaf tissues during a dehydration treatment indicated that all three transgenic lines had lower water loss rates than WT plants, especially when the detached leaves were exposed to dehydration for 3 h or longer (Figure 2D). These findings demonstrated that the transgenic plants had better water retention ability under water scarcity conditions. In a previous study, improved drought tolerance associated with reduced water transpiration rates of transgenic *Arabidopsis* plants ectopically expressing chickpea (*Cicer arietinum*) *CarNAC4* has also been reported [73]. Furthermore, transgenic *Arabidopsis* harboring the maize *ZmNAC55* showed better drought tolerance, which was partially attributed to the faster stomatal closure rates, leading to a slower cellular dehydration process under water-deficit conditions [74]. In another research, the enhanced tolerance of *TaNAC2*-transgenic *Arabidopsis* lines to drought was also featured with a lower water loss rate in detached leaves coupled with lower osmotic potential [75].

### 2.3. GmNAC019-Transgenic Plants Exhibited Decreased Drought-Induced Oxidative Stress by Regulating ROS Metabolism

Under the shortage of water availability, oxidative stress is triggered as a secondary stress in plants due to the accumulation of intracellular ROS [76,77]. Therefore, evaluation of the hydrogen peroxide (H_2_O_2_) contents (i.e., one of commonly accumulated ROS) and activities of key antioxidant enzymes are considered important in estimating the drought-induced oxidative stress levels and plant resistance capacities [50,61,69].

**Figure 3:**
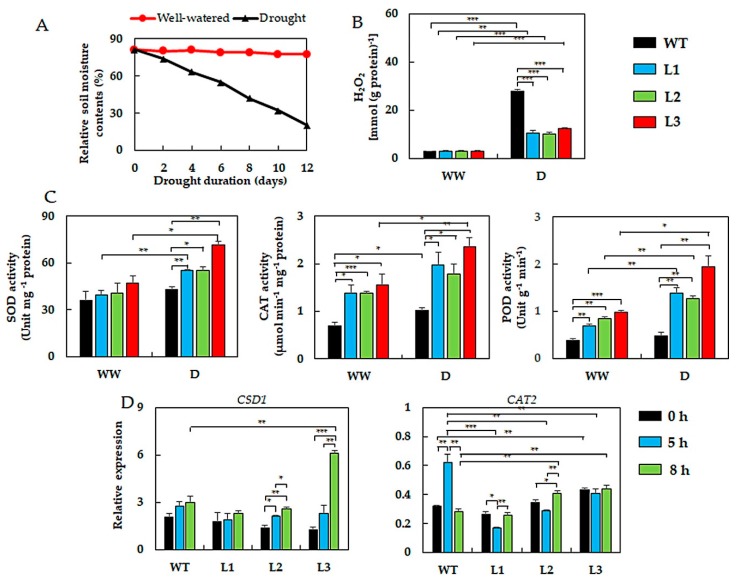
Cellular hydrogen peroxide (H_2_O_2_) content, and antioxidant defense-related enzyme activities and gene expression in *GmNAC019*-transgenic *Arabidopsis* (L1, L2, L3) and wild-type (WT) plants under normal and water-stressed conditions. (**A**) Monitored relative soil moisture content during a 12-day period of soil-drying (*n* = 6). (**B**) Cellular H_2_O_2_ content (*n* = 3) with irrigation (WW) or without irrigation (D) for 12 days. (**C**) Activities of superoxide dismutase (SOD), catalase (CAT) and peroxidase (POD) enzymes with irrigation or without irrigation for 12 days (*n* = 3). (**D**) Expression pattern of *CSD1* (*copper/zinc superoxide dismutase 1*) and *CAT2* (*catalase 2*) genes in plants subjected to 0, 5 and 8 h of dehydration (*n* = 3). The drought and dehydration treatments were applied to four-week-old and three-week-old plants, respectively. Error bars represent SEs. Asterisks indicate significant differences (* *p*-value < 0.05; ** *p*-value < 0.01; *** *p*-value < 0.001) between each transgenic line and WT plants grown under the same conditions, and within the same genotype exposed to two treatment conditions, according to a Student’s *t*-test.

Our data revealed that under normal growth conditions, there were no distinct differences in endogenous H_2_O_2_ levels across all genotypes, and the H_2_O_2_ concentrations detected were relatively low (Figure 3A,B). However, in plants experiencing drought, the H_2_O_2_ levels increased by 3 to 4-fold in the transgenic plants and around 10-fold in the WT plants in comparison with irrigated corresponding plants (Figure 3B). This result indicated that during the drought, the WT plants were likely exposed to more serious oxidative stress than the transgenic lines. Next, we determined the activities of three ROS-scavenging marker enzymes, including superoxide dismutase (SOD), peroxidase (POD) and catalase (CAT) [78,79]. Under water deficit conditions, while the WT plants showed slightly enhanced levels of SOD, the transgenic lines, especially L3, showed even more highly increased SOD activities (Figure 3C). Enhanced SOD activities suggested a better capacity in scavenging superoxide (the substrate of SOD) in the transgenic lines than in WT plants. Regarding the CAT and POD, higher enzyme activities in the transgenic plants were observed not only under drought but also normal growth conditions, indicating that the accumulation of H_2_O_2_ in these transgenic plants could also be prevented by their stronger activities. It is worth mentioning that under normal irrigation, while H_2_O_2_ levels were comparable in all genotypes, the CAT and POD activities were higher in the transgenic lines than WT plants (Figure 3B,C), which might indicate an acclimation state ready for faster increases of these enzymes in transgenic plants to better prevent H_2_O_2_ accumulation when drought occurs, rather for reducing the basal H_2_O_2_ levels produced under non-stressed conditions that are required for normal plant development as an important signaling molecule [79,80,81]. Noticeable increases in activities of these antioxidant enzymes under normal growth conditions were also observed in other studies using different kinds of TFs, including the ectopic expression of *Pyrus betulifolia PbeNAC1* in tobacco [82] and of *Vitis labrusca* × *V. vinifera VlWRKY3* in *A. thaliana* [83].

To gain an insight into the molecular basis of GmNAC019 function in plant drought tolerance in relation to ROS-removing enzymes, the expression levels of *CSD1* (*copper/zinc superoxide dismutase 1*) and *CAT2* (*catalase 2*) genes in transgenic and WT plants were compared. These genes encode the SOD [84] and CAT [85] enzymes, respectively. To examine how gene expression was altered under different durations of water-deficit conditions, the plants were subjected to a dehydration treatment that resulted in mild and severe cellular dehydration conditions, similar to what could be observed under short-term drought (i.e., under short-term dehydration) and long-term drought (i.e., under prolonged dehydration), respectively. According to our expression analysis, *CSD1* showed a significant upregulation in transgenic lines L2 and L3 after 8-h dehydration treatment, with pronounced induction observed in L3 (nearly 5-fold) (Figure 3D). This result suggested that the upregulation of *CDS1* gene in the transgenic plants was dependent on the transcript levels of *GmNAC019* gene, as L3 showed more than 2-fold higher *GmNAC019* expression than did the L1 and L2 (Figure 1A). Regarding the *CAT2* expression, three distinct transcript patterns of *CAT2* were noticed over the course of dehydration treatment (Figure 3D). In the WT plants, *CAT2* expression reached the peak at 5 h of dehydration, and as followed by a drop to the base level 3 h later. Meanwhile, the transgenic L1 and L2 lines displayed an inverted expression profile; i.e. they showed a slight decrease after 5 h, but an increase after 8 h of dehydration in *CAT2* transcript abundance. On the other hand, line L3 showed stable *CAT2* expression over the dehydration period, and notably displayed higher *CAT2* transcript level than WT did after 8-h dehydration treatment. Therefore, examination of the *CAT2* expression in an extended dehydration would be useful to clarify its response to dehydration. It should also be mentioned that gene expression and corresponding enzyme activity levels were not very well correlated in some cases upon the water-stress treatments (e.g., *CSD1* transcript levels versus SOD enzyme activity levels in L1 and L2, and *CAT2* transcript levels versus CAT enzyme activity levels in L3) (Figure 3C,D). Such inconsistencies could be explained by the fact that the analysis of enzymatic activities was based on the performance of all forms of isozymes/isoforms, while the analysis of gene expression was only for one gene encoding a particular isozyme/isoform [86]. Therefore, expression of other *CSD1* and *CAT2* homologous genes should be worthily studied for acquiring a clearer picture.

Previously, a great number of studies reported the successful improvements of drought resistance in plants by overexpressing/ectopically expressing various *NAC* genes. For example, ectopic expression of *TaNac29* led to enhanced drought tolerance in transgenic *Arabidopsis*, partly due to the increase in detoxifying activities of SOD and CAT, and the reduction in H_2_O_2_ accumulation [50]. Likewise, *Arabidopsis* plants harboring the soybean *GmNAC085* expression also displayed better adaptation to drought conditions. Altered features observed in these transgenic lines included lower transpiration rate and stronger ROS-scavenging activities [69]. Transgenic tomato (*Solanum lycopersicum*) plants harboring tomato transgene *SlNAC35* [61], and transgenic rice carrying the rice transgene *ONAC095* [60] also conferred improved drought resistance by acquiring similar modified attributes. Therefore, the findings imply that the *GmNAC019*-transgenic plants possessed an improved enzymatic antioxidant defense system to more efficiently scavenge drought-induced ROS, which enabled them to survive better under the adverse water scarcity.

### 2.4. GmNAC019-Transgenic Plants Were More Sensitive to ABA

**Figure 4:**
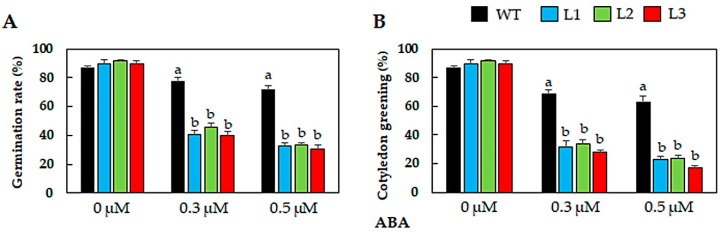
The effects of ABA application on seed germination and cotyledon development of *GmNAC019*-transgenic (L1, L2 and L3) versus wild-type (WT) plants. (**A**) Germination rates of seeds that were recorded after three days of growth on MS medium supplied with or without ABA (*n* = 3). (**B**) Cotyledon greening rates that were recorded after seven days of growth on MS medium supplied with or without ABA (*n* = 3). Error bars represent SEs. Different letters indicate significant differences (*p*-value < 0.05) among the genotypes under the same treatment as analyzed by a Duncan’s multiple range test.

It is known that ABA is an important phytohormone that regulates plant responses to various abiotic stresses, particularly drought [87]. To find out if GmNAC019 would act in plant drought responses through an ABA-mediated pathway, we studied the effects of this phytohormone on seed germination and cotyledon greening of the transgenic plants. Results showed that supplementation of exogenous ABA to the medium resulted in more remarkably reduced germination rates of the seeds of transgenic lines than WT plants (by 46–50% and 57–60% in transgenic lines versus 10–16% in WT at 0.3 and 0.5 µM ABA, respectively) (Figure 4A). In support of the germination data, a lower proportion of transgenic plants could develop cotyledons (Figure 4B). In detail, under 0.3 µM ABA conditions, 79% WT plants could display greening cotyledon, whereas only 30–39% of transgenic plants depending on each genotype could show this phenotype. At the higher 0.5 µM ABA concentration, the values were 72% in WT and 18–31% in the transgenic lines. The ABA hypersensitivity observed in the *GmNAC019*-transgenic lines indicates that GmNAC019 participates in regulation of plant response to drought in an ABA-dependent manner.

It is known that both ABA-dependent and ABA-independent pathways play vital roles in mediating plant responses to adverse environmental conditions [76]. NAC TFs have been found to be involved in both pathways in relation to plant drought responses, suggesting their diverse actions in drought tolerance [88,89]. Similar to our findings, in the study of Yang et al. [90], the drought-tolerant *Arabidopsis* plants ectopically expressing the *Miscanthus lutarioriparius MlNAC5* were shown to be highly sensitive to ABA treatment during the post-germination stage. Functional analyses of TaNAC29 [50], ZmSNAC1 [51], AhNAC2 [91] and OsNAC52 [92] in *Arabidopsis* also revealed that these TFs act as positive regulators for plant responses to dehydration or drought in the ABA-dependent signaling pathway. It is suggested that this ABA-responsive feature of the examined NACs may enhance the stomatal closure, and thus, reduce water transpiration from leaves [73,74]. On the other hand, the foxtail millet (*Setaria italica*) SiNAC110 has been demonstrated to exhibit ABA-independent actions in enhancing the drought tolerance of *SiNAC110*-transgenic *Arabidopsis* plants [93].

## 3. Materials and Methods

### 3.1. Plant Materials and Growing Conditions

*Arabidopsis* ecotype Col-0 was used in all experiments. The procedure for cloning of the complete coding sequence of *GmNAC019* into pGHX vector under the control of the *CaMV 35S* promoter was similar to pGKX in previous studies [69,94], except using hygromycin instead of kanamycin for selection of transformed plants. The transgenic *Arabidopsis* plants harboring *CaMV35S:GmNAC019* were generated by *Agrobacterium*-mediated floral dipping approach [95]. Homozygous progenies were identified by monitoring three consecutive *Arabidopsis* generations based on resistance to hygromycin (15 mg/L) and Mendelian segregation analyses [96]. Three independent transgenic lines were identified by this approach, and the *GmNAC019* expression levels in rosette leaves of four-week-old plants (three biological replicates per genotype) were analyzed by RT-qPCR using the *GmNAC019*-specific primers (Supplementary Table S1).

In all experiments performed in this study, the seeds were surface-sterilized prior to germination. The information for seed sterilization, germination and growing condition was described in a previously published study [70]. When the plants were transferred to soil for an assay, the relative soil moisture contents (SMCs) were regularly monitored, with the aid of a Moisture Meter (TK-100G, Yieryi, Guangdong, China), by measuring spontaneously six different positions per time point to obtain the average SMC values.

### 3.2. Phenotypic Evaluation of Transgenic Plants under Non-Stressed Conditions

Four-week-old seedlings (two weeks on Murashige and Skoog (MS) plates followed by two weeks on soil) grown under normal conditions were used to assess the growth and development. The plants were removed from soil for recording their tap root length. The longest leaf of individual plants was measured to calculate the maximum rosette radius according to a previous study [69] using the Image-J software (https://imagej.nih.gov/ij/). Rosette area was also recorded following the described procedure [97] using the PhotoshopCC 2019 (Adobe, San José, California, USA). Ten plants of each genotype were used for these analyses.

### 3.3. Evaluation of Drought Survival Rate

Survival rate upon drought exposure was determined following the same-tray method in which the wild-type (WT) and transgenic plants were grown on the same trays [70]. In brief, two-week-old seedlings were transferred from MS plates to soil. The plants were allowed to grow for two weeks under normal watering conditions before they were subjected to non-irrigation for 12 days. After this period, water was re-applied for the next three days before the percentage of recovered plants per genotype was evaluated. Photographs were taken before the drought treatments and after three-day recovery. The assay was conducted using three biological replicates per genotype, with 20 plants for each replicate.

### 3.4. Measurement of Water Loss Rate

To evaluate the water loss rate, two largest leaves from each four-week-old seedling were dissected and immediately weighed (Sartorius, number 68285437, Göttingen, Germany) to obtain initial fresh weight (FW) of each leaf. The harvested leaves were then placed on a laboratory bench for gradual air-dehydration during 5 h, and their weights were recorded every 0.5 h during the examined period. The water loss rate was estimated based on percentage of FW reduction over designated time points. Nine plants of each genotype were used to perform this assay, and the average weight of two leaves dissected from the same plant was used as the replicate sample [98].

### 3.5. Measurement of Cellular H_2_O_2_ and Activities of Antioxidant Enzymes

Four-week-old plants of all genotypes were subjected to a water withholding treatment for 12 days before the leaf tissues (0.2 g per plant for each replicate, three replicates per genotype) were collected for measurement. H_2_O_2_ content was determined following the previous method [99] with minor modifications according to Nguyen et al. [70]. For enzymatic activity analyses, 0.2 g leaves were ground in 2 mL of pre-chilled extraction buffer (potassium phosphate buffer 1 M, EDTA 0.1 M and 2% polyvinylpyrrolidone (molecular weight 8000), pH 7.8). Following centrifugation, the supernatant was used for total soluble protein content determination by Bradford method [100], and superoxide dismutase [101], peroxidase [102] and catalase [103] activity assays using the published procedures.

### 3.6. Expression Analyses of Selected Drought-Responsive Genes

Three-week-old plants were subjected to dehydration on bench for 5 and 8 h before the whole treated plants were collected and frozen in liquid nitrogen. Purified total RNA was obtained from these samples by using GeneJET Plant RNA Purification Kit (Thermo Scientific, Waltham, MA, USA) and RapidOut DNA Removal Kit (Thermo Scientific, Waltham, MA, USA). For cDNA synthesis, 1 µg of total RNA and Maxima First Strand cDNA Synthesis Kit (Thermo Scientific, Waltham, MA, USA) were used. The procedure for RT-qPCR was conducted according to previously described study [67], with *Actin2* being used as internal reference gene [104]. Sequences of specific primers for reference and stress-related target genes are listed in Supplementary Table S1. Comparative expression analysis was performed by using the 2^−ΔΔCt^ method [105]. Three biological replicates from each genotype were used in each treatment condition in this assay.

### 3.7. Evaluation of Exogenous ABA Application to Seed Germination and Cotyledon Development

To study the effect of ABA on germination, *Arabidopsis* seeds were sown on MS medium supplemented with 0, 0.3 and 0.5 µM ABA for three days, before the germination rate was recorded with reference to seed radicle appearance [106]. A preparation similar to the germination assay was also performed for examining the effect of ABA on cotyledon greening after seven days of incubation, by calculating the proportion of seedlings displaying green cotyledon [106]. The plates were kept under non-stressed growth conditions [70]. For each assay, three sets of replicates of each genotype, with the size of 100 seeds per replicate, were analyzed.

### 3.8. Statistical Analysis

Student’s *t*-test and Duncan’s multiple range test from one-way analysis of variance (ANOVA) were used for comparisons between two examined populations and among three populations or more, respectively, to determine whether the differences were statistically significant with *p*-values < 0.05.

## 4. Conclusions

This work investigated the biological functions of the soybean GmNAC019 in plant response to drought using the transgenic *Arabidopsis* plants ectopically expressing this gene. The findings demonstrated that this TF acts as a positive regulator to mediate plant drought tolerance in an ABA-dependent manner, particularly via regulating plant transpiration process and enzymatic antioxidant defense.

## Supplementary Materials

Supplementary materials can be found at https://www.mdpi.com/1422-0067/21/1/286/s1. Table S1: Genes and primers used for RT-qPCR analysis.

## Abbreviations

ABA	Abscisic acid
CAT	Catalase
H_2_O_2_	Hydrogen peroxide
NAC	NAM, ATAF1/2, CUC2
POD	Peroxidase
ROS	Reactive oxygen species
SOD	Superoxide dismutase
TF	Transcription factor

## References

[1] Yadav N.S., Shukla P.S., Jha A., Agarwal P.K., Jha B. (2012). The *SbSOS1* gene from the extreme halophyte *Salicornia brachiata* enhances Na^+^ loading in xylem and confers salt tolerance in transgenic tobacco. BMC Plant Biol..

[2] Hoang X.L.T., Nhi D.N.H., Thu N.B.A., Thao N.P., Tran L.-S.P. (2017). Transcription factors and their roles in signal transduction in plants under abiotic stresses. Curr. Genom..

[3] Zhu J.-K. (2016). Abiotic stress signaling and responses in plants. Cell.

[4] He M., He C.-Q., Ding N.-Z. (2018). Abiotic stresses: General defenses of land plants and chances for engineering multistress tolerance. Front. Plant Sci..

[5] Tran L.-S.P., Quach T.N., Guttikonda S.K., Aldrich D.L., Kumar R., Neelakandan A., Valliyodan B., Nguyen H.T. (2009). Molecular characterization of stress–inducible *GmNAC* genes in soybean. Mol. Genet. Genom..

[6] Baillo E.H., Kimotho R.N., Zhang Z., Xu P. (2019). Transcription factors associated with abiotic and biotic stress tolerance and their potential for crops improvement. Genes.

[7] Xie Z., Nolan T.M., Jiang H., Yin Y. (2019). AP2/ERF transcription factor regulatory networks in hormone and abiotic stress responses in *Arabidopsis*. Front. Plant Sci..

[8] Srivastava R., Kumar R. (2019). The expanding roles of APETALA2/Ethylene Responsive Factors and their potential applications in crop improvement. Brief. Funct. Genom..

[9] Marques D.N., Reis S.P.D., de Souza C.R.B. (2017). Plant NAC transcription factors responsive to abiotic stresses. Plant Gene.

[10] Tweneboah S., Oh S.-K. (2017). Biological roles of NAC transcription factors in the regulation of biotic and abiotic stress responses in solanaceous crops. J. Plant Biotechnol..

[11] Noman A., Liu Z., Aqeel M., Zainab M., Khan M.I., Hussain A., Ashraf M.F., Li X., Weng Y., He S. (2017). Basic leucine zipper domain transcription factors: The vanguards in plant immunity. Biotechnol. Lett..

[12] Banerjee A., Roychoudhury A. (2017). Abscisic–acid–dependent basic leucine zipper (bZIP) transcription factors in plant abiotic stress. Protoplasma.

[13] Ambawat S., Sharma P., Yadav N.R., Yadav R.C. (2013). MYB transcription factor genes as regulators for plant responses: An overview. Physiol. Mol. Biol. Plants.

[14] Erpen L., Devi H.S., Grosser J.W., Dutt M. (2018). Potential use of the DREB/ERF, MYB, NAC and WRKY transcription factors to improve abiotic and biotic stress in transgenic plants. Plant Cell Tiss. Org. Cult..

[15] Jiang J., Ma S., Ye N., Jiang M., Cao J., Zhang J. (2017). WRKY transcription factors in plant responses to stresses. J. Integr. Plant Biol..

[16] Bai Y., Sunarti S., Kissoudis C., Visser R.G.F., van der Linden C.G. (2018). The role of tomato *WRKY* genes in plant responses to combined abiotic and biotic stresses. Front. Plant Sci..

[17] Wang K., Ding Y., Cai C., Chen Z., Zhu C. (2019). The role of C_2_H_2_ zinc finger proteins in plant responses to abiotic stresses. Physiol. Plant..

[18] Li W.T., He M., Wang J., Wang Y.P. (2013). Zinc finger protein (ZFP) in plants—A review. Plant OMICS.

[19] Kiełbowicz-Matuk A. (2012). Involvement of plant C_2_H_2_—Type zinc finger transcription factors in stress responses. Plant Sci..

[20] Takada S., Hibara K., Ishida T., Tasaka M. (2001). The *CUP*–*SHAPED COTYLEDON1* gene of *Arabidopsis* regulates shoot apical meristem formation. Development.

[21] Larsson E., Sundström J.F., Sitbon F., von Arnold S. (2012). Expression of *PaNAC01*, a *Picea abies CUP*–*SHAPED COTYLEDON* orthologue, is regulated by polar auxin transport and associated with differentiation of the shoot apical meristem and formation of separated cotyledons. Ann. Bot..

[22] Wang F., Lin R., Feng J., Chen W., Qiu D., Xu S. (2015). TaNAC1 acts as a negative regulator of stripe rust resistance in wheat, enhances susceptibility to *Pseudomonas syringae*, and promotes lateral root development in transgenic *Arabidopsis thaliana*. Front. Plant Sci..

[23] Willemsen V., Bauch M., Bennett T., Campilho A., Wolkenfelt H., Xu J., Haseloff J., Scheres B. (2008). The NAC domain transcription factors FEZ and SOMBRERO control the orientation of cell division plane in *Arabidopsis* root stem cells. Dev. Cell..

[24] Souer E., van Houwelingen A., Kloos D., Mol J., Koes R. (1996). The *no apical meristem* gene of Petunia is required for pattern formation in embryos and flowers and is expressed at meristem and primordia boundaries. Cell.

[25] Mitsuda N., Seki M., Shinozaki K., Ohme-Takagi M. (2005). The NAC transcription factors NST1 and NST2 of *Arabidopsis* regulate secondary wall thickenings and are required for anther dehiscence. Plant Cell.

[26] Mitsuda N., Iwase A., Yamamoto H., Yoshida M., Seki M., Shinozaki K., Ohme-Takagi M. (2007). NAC transcription factors, NST1 and NST3, are key regulators of the formation of secondary walls in woody tissues of *Arabidopsis*. Plant Cell.

[27] Yang Q., Zhang H., Liu C., Huang L., Zhao L., Zhang A. (2018). A NAC Transcription factor ZmNAC84 affects pollen development through the repression of *ZmRbohH* expression in maize. J. Plant Biol..

[28] Guo S., Dai S., Singh P.K., Wang H., Wang Y., Tan J.L.H., Wee W., Ito T. (2018). A membrane–bound NAC–like transcription factor OsNTL5 represses the flowering in *Oryza sativa*. Front. Plant Sci..

[29] Sun H., Hu M., Li J., Chen L., Li M., Zhang S., Zhang X., Yang X. (2018). Comprehensive analysis of NAC transcription factors uncovers their roles during fiber development and stress response in cotton. BMC Plant Biol..

[30] Liu X., Wang T., Bartholomew E., Black K., Dong M., Zhang Y., Yang S., Cai Y., Xue S., Weng Y. (2018). Comprehensive analysis of NAC transcription factors and their expression during fruit spine development in cucumber (*Cucumis sativus* L.). Hortic. Res..

[31] Tian H., Wang X., Guo H., Cheng Y., Hou C., Chen J.-G., Wang S. (2017). NTL8 regulates trichome formation in *Arabidopsis* by directly activating R_3_ MYB genes *TRY* and *TCL1*. Plant Physiol..

[32] Gao Y., Wei W., Zhao X., Tan X., Fan Z., Zhang Y., Jing Y., Meng L., Zhu B., Zhu H. (2018). A NAC transcription factor, NOR–like1, is a new positive regulator of tomato fruit ripening. Hortic. Res..

[33] Kim H.J., Nam H.G., Lim P.O. (2016). Regulatory network of NAC transcription factors in leaf senescence. Curr. Opin. Plant Biol..

[34] Fujita M., Fujita Y., Maruyama K., Seki M., Hiratsu K., Ohme-Takagi M., Tran L.-S.P., Yamaguchi-Shinozaki K., Shinozaki K. (2004). A dehydration–induced NAC protein, RD26, is involved in a novel ABA–dependent stress–signaling pathway. Plant J..

[35] Bu Q., Jiang H., Li C.-B., Zhai Q., Zhang J., Wu X., Sun J., Xie Q., Li C. (2008). Role of the *Arabidopsis thaliana* NAC transcription factors ANAC019 and ANAC055 in regulating jasmonic acid–signaled defense responses. Cell Res..

[36] Yoshii M., Yamazaki M., Rakwal R., Kishi-Kaboshi M., Miyao A., Hirochika H. (2010). The NAC transcription factor RIM1 of rice is a new regulator of jasmonate signaling. Plant J..

[37] Xie Q., Frugis G., Colgan D., Chua N.H. (2000). *Arabidopsis* NAC1 transduces auxin signal downstream of TIR1 to promote lateral root development. Genes Dev..

[38] Huh S.U., Lee S.-B., Kim H.H., Paek K.-H. (2012). ATAF2, a NAC transcription factor, binds to the promoter and regulates *NIT2* gene expression involved in auxin biosynthesis. Mol. Cells.

[39] Pei H., Ma N., Tian J., Luo J., Chen J., Li J., Zheng Y., Chen X., Fei Z., Gao J. (2013). An NAC transcription factor controls ethylene–regulated cell expansion in flower petals. Plant Physiol..

[40] Kim H.J., Hong S.H., Kim Y.W., Lee I.H., Jun J.H., Phee B.-K., Rupak T., Jeong H., Lee Y., Hong B.S. (2014). Gene regulatory cascade of senescence–associated NAC transcription factors activated by ETHYLENE–INSENSITIVE2–mediated leaf senescence signalling in *Arabidopsis*. J. Exp. Bot..

[41] Chen X., Lu S., Wang Y., Zhang X., Lv B., Luo L., Xi D., Shen J., Ma H., Ming F. (2015). *OsNAC2* encoding a NAC transcription factor that affects plant height through mediating the gibberellic acid pathway in rice. Plant J..

[42] Fang Y., You J., Xie K., Xie W., Xiong L. (2008). Systematic sequence analysis and identification of tissue–specific or stress–responsive genes of NAC transcription factor family in rice. Mol. Genet. Genom..

[43] Le D.T., Nishiyama R., Watanabe Y., Mochida K., Yamaguchi-Shinozaki K., Shinozaki K., Tran L.-S.P. (2011). Genome–wide survey and expression analysis of the plant–specific NAC transcription factor family in soybean during development and dehydration stress. DNA Res..

[44] Greve K., La Cour T., Jensen M.K., Poulsen F.M., Skriver K. (2003). Interactions between plant RING–H2 and plant–specific NAC (NAM/ATAF1/2/CUC2) proteins: RING–H2 molecular specificity and cellular localization. Biochem. J..

[45] Jensen M.K., Kjaersgaard T., Nielsen M.M., Galberg P., Petersen K., O’Shea C., Skriver K. (2010). The *Arabidopsis thaliana* NAC transcription factor family: Structure–function relationships and determinants of ANAC019 stress signalling. Biochem. J..

[46] O’Shea C., Kryger M., Stender E.G.P., Kragelund B.B., Willemoës M., Skriver K. (2015). Protein intrinsic disorder in *Arabidopsis* NAC transcription factors: Transcriptional activation by ANAC013 and ANAC046 and their interactions with RCD1. Biochem. J..

[47] Chen Q., Wang Q., Xiong L., Lou Z. (2011). A structural view of the conserved domain of rice stress–responsive NAC1. Protein Cell.

[48] Tran L.-S.P., Nakashima K., Sakuma Y., Osakabe Y., Qin F., Simpson S.D., Maruyama K., Fujita Y., Shinozaki K., Yamaguchi-Shinozaki K. (2007). Co–expression of the stress–inducible zinc finger homeodomain ZFHD1 and NAC transcription factors enhances expression of the *ERD1* gene in *Arabidopsis*. Plant J..

[49] Tran L.-S.P., Nakashima K., Sakuma Y., Simpson S.D., Fujita Y., Maruyama K., Fujita M., Seki M., Shinozaki K., Yamaguchi-Shinozaki K. (2004). Isolation and functional analysis of *Arabidopsis* stress–inducible NAC transcription factors that bind to a drought–responsive *cis*–element in the *early responsive to dehydration stress 1* promoter. Plant Cell.

[50] Huang Q., Wang Y., Li B., Chang J., Chen M., Li K., Yang G., He G. (2015). TaNAC29, a NAC transcription factor from wheat, enhances salt and drought tolerance in transgenic *Arabidopsis*. BMC Plant Biol..

[51] Lu M., Ying S., Zhang D.-F., Shi Y.-S., Song Y.-C., Wang T.-Y., Li Y. (2012). A maize stress–responsive NAC transcription factor, ZmSNAC1, confers enhanced tolerance to dehydration in transgenic *Arabidopsis*. Plant Cell Rep..

[52] Hu H., Dai M., Yao J., Xiao B., Li X., Zhang Q., Xiong L. (2006). Overexpressing a NAM, ATAF, and CUC (NAC) transcription factor enhances drought resistance and salt tolerance in rice. Proc. Natl. Acad. Sci. USA.

[53] Hong Y., Zhang H., Huang L., Li D., Song F. (2016). Overexpression of a stress–responsive NAC transcription factor gene *ONAC022* improves drought and salt tolerance in rice. Front. Plant Sci..

[54] Lee D.-K., Chung P.J., Jeong J.S., Jang G., Bang S.W., Jung H., Kim Y.S., Ha S.-H., Choi Y.D., Kim J.-K. (2017). The rice OsNAC6 transcription factor orchestrates multiple molecular mechanisms involving root structural adaptions and nicotianamine biosynthesis for drought tolerance. Plant Biotechnol. J..

[55] Fang Y., Liao K., Du H., Xu Y., Song H., Li X., Xiong L. (2015). A stress–responsive NAC transcription factor SNAC3 confers heat and drought tolerance through modulation of reactive oxygen species in rice. J. Exp. Bot..

[56] Liu X., Liu S., Wu J., Zhang B., Li X., Yan Y., Li L. (2013). Overexpression of *Arachis hypogaea NAC3* in tobacco enhances dehydration and drought tolerance by increasing superoxide scavenging. Plant Physiol. Biochem..

[57] Tang Y., Liu M., Gao S., Zhang Z., Zhao X., Zhao C., Zhang F., Chen X. (2012). Molecular characterization of novel *TaNAC* genes in wheat and overexpression of *TaNAC2a* confers drought tolerance in tobacco. Physiol. Plant..

[58] Mao X., Chen S., Li A., Zhai C., Jing R. (2014). Novel NAC transcription factor TaNAC67 confers enhanced multi–abiotic stress tolerances in *Arabidopsis*. PLoS ONE.

[59] Pandurangaiah M., Lokanadha Rao G., Sudhakarbabu O., Nareshkumar A., Kiranmai K., Lokesh U., Thapa G., Sudhakar C. (2014). Overexpression of horsegram (*Macrotyloma uniflorum* Lam.Verdc.) NAC transcriptional factor (*MuNAC4*) in groundnut confers enhanced drought tolerance. Mol. Biotechnol..

[60] Huang L., Hong Y., Zhang H., Li D., Song F. (2016). Rice NAC transcription factor ONAC095 plays opposite roles in drought and cold stress tolerance. BMC Plant Biol..

[61] Wang G., Zhang S., Ma X., Wang Y., Kong F., Meng Q. (2016). A stress–associated NAC transcription factor (SlNAC35) from tomato plays a positive role in biotic and abiotic stresses. Physiol. Plant..

[62] Chung P.J., Jung H., Choi Y.D., Kim J.-K. (2018). Genome–wide analyses of direct target genes of four rice NAC–domain transcription factors involved in drought tolerance. BMC Genom..

[63] Abdelrahman M., El-Sayed M., Jogaiah S., Burritt D.J., Tran L.-S.P. (2017). The “STAY–GREEN” trait and phytohormone signaling networks in plants under heat stress. Plant Cell Rep..

[64] Patil M., Ramu S.V., Jathish P., Sreevathsa R., Reddy P.C., Prasad T.G., Udayakumar M. (2014). Overexpression of *AtNAC2* (*ANAC092*) in groundnut (*Arachis hypogaea* L.) improves abiotic stress tolerance. Plant Biotechnol. Rep..

[65] Jeong J.S., Kim Y.S., Baek K.H., Jung H., Ha S.-H., Do Choi Y., Kim M., Reuzeau C., Kim J.-K. (2010). Root–specific expression of *OsNAC10* improves drought tolerance and grain yield in rice under field drought conditions. Plant Physiol..

[66] Hussain R.M., Ali M., Feng X., Li X. (2017). The essence of *NAC* gene family to the cultivation of drought–resistant soybean (*Glycine max* L. Merr.) cultivars. BMC Plant Biol..

[67] Thao N.P., Thu N.B.A., Hoang X.L.T., Van Ha C., Tran L.-S.P. (2013). Differential expression analysis of a subset of drought–responsive *GmNAC* genes in two soybean cultivars differing in drought tolerance. Int. J. Mol. Sci..

[68] Thu N.B.A., Hoang X.L.T., Doan H., Nguyen T.-H., Bui D., Thao N.P., Phan Tran L.-S. (2014). Differential expression analysis of a subset of *GmNAC* genes in shoots of two contrasting drought–responsive soybean cultivars DT51 and MTD720 under normal and drought conditions. Mol. Biol. Rep..

[69] Nguyen K.H., Mostofa M.G., Li W., Van Ha C., Watanabe Y., Le D.T., Thao N.P., Tran L.-S.P. (2018). The soybean transcription factor GmNAC085 enhances drought tolerance in *Arabidopsis*. Environ. Exp. Bot..

[70] Nguyen N.C., Hoang X.L.T., Quang N.T., Binh N.X., Watanabe Y., Thao N.P., Tran L.-S.P. (2019). Ectopic expression of *Glycine max GmNAC109* enhances drought tolerance and ABA sensitivity in *Arabidopsis*. Biomolecules.

[71] Liu C., Wang B., Li Z., Peng Z., Zhang J. (2018). TsNAC1 is a key transcription factor in abiotic stress resistance and growth. Plant Physiol..

[72] Kato H., Motomura T., Komeda Y., Saito T., Kato A. (2010). Overexpression of the NAC transcription factor family gene *ANAC036* results in a dwarf phenotype in *Arabidopsis thaliana*. J. Plant Physiol..

[73] Yu X., Liu Y., Wang S., Tao Y., Wang Z., Shu Y., Peng H., Mijiti A., Wang Z., Zhang H. (2016). CarNAC4, a NAC–type chickpea transcription factor conferring enhanced drought and salt stress tolerances in *Arabidopsis*. Plant Cell Rep..

[74] Mao H., Yu L., Han R., Li Z., Liu H. (2016). ZmNAC55, a maize stress–responsive NAC transcription factor, confers drought resistance in transgenic *Arabidopsis*. Plant Physiol. Biochem..

[75] Mao X., Zhang H., Qian X., Li A., Zhao G., Jing R. (2012). *TaNAC2*, a NAC–type wheat transcription factor conferring enhanced multiple abiotic stress tolerances in *Arabidopsis*. J. Exp. Bot..

[76] Hoang X.L.T., Thu N.B.A., Thao N.P., Tran L.-S.P., Ahmad P., Wani M.R., Azooz M.M., Tran L.-S.P. (2014). Transcription factors in abiotic stress responses: Their potentials in crop improvement. Improvement of Crops in the Era of Climatic Changes.

[77] Sharma P., Jha A.B., Dubey R.S., Pessarakli M. (2012). Reactive oxygen species, oxidative damage, and antioxidative defense mechanism in plants under stressful conditions. J. Bot..

[78] Bowler C., Montagu M.V., Inze D. (1992). Superoxide dismutase and stress tolerance. Ann. Rev. Plant Physiol. Plant Mol. Biol..

[79] Hossain M.A., Bhattacharjee S., Armin S.-M., Qian P., Xin W., Li H.-Y., Burritt D.J., Fujita M., Tran L.-S.P. (2015). Hydrogen peroxide priming modulates abiotic oxidative stress tolerance: Insights from ROS detoxification and scavenging. Front. Plant Sci..

[80] Mittler R. (2017). ROS are good. Trends Plant Sci..

[81] Waszczak C., Carmody M., Kangasjärvi J. (2018). Reactive oxygen species in plant signaling. Ann. Rev. Plant Biol..

[82] Jin C., Li K.-Q., Xu X.-Y., Zhang H.-P., Chen H.-X., Chen Y.-H., Hao J., Wang Y., Huang X.-S., Zhang S.-L. (2017). A novel NAC transcription factor, *PbeNAC1*, of *Pyrus betulifolia* confers cold and drought tolerance via interacting with *PbeDREBs* and activating the expression of stress–responsive genes. Front. Plant Sci..

[83] Guo R., Qiao H., Zhao J., Wang X., Tu M., Guo C., Wan R., Li Z., Wang X. (2018). The grape *VlWRKY3* gene promotes abiotic and biotic stress tolerance in transgenic *Arabidopsis thaliana*. Front. Plant Sci..

[84] Chen Y., Jiang J., Song A., Chen S., Shan H., Luo H., Gu C., Sun J., Zhu L., Fang W. (2013). Ambient temperature enhanced freezing tolerance of *Chrysanthemum dichrum CdICE1 Arabidopsis* via *miR398*. BMC Biol..

[85] Du Y.-Y., Wang P.-C., Chen J., Song C.-P. (2008). Comprehensive functional analysis of the catalase gene family in *Arabidopsis thaliana*. J. Integr. Plant Biol..

[86] Harb A., Awad D., Samarah N. (2015). Gene expression and activity of antioxidant enzymes in barley (*Hordeum vulgare* L.) under controlled severe drought. J. Plant Interact..

[87] Osakabe Y., Yamaguchi-Shinozaki K., Shinozaki K., Tran L.-S.P. (2014). ABA control of plant macroelement membrane transport systems in response to water deficit and high salinity. New Phytol..

[88] Puranik S., Sahu P., Srivastava P., Prasad M. (2012). NAC proteins: Regulation and role in stress tolerance. Trends Plant Sci..

[89] Ha C.V., Nasr E.M., Watanabe Y., Tran U.T., Sulieman S., Mochida K., Nguyen V.D., Tran L.-S.P. (2014). Genome–wide identification and expression analysis of the *CaNAC* family members in chickpea during development, dehydration and ABA treatments. PLoS ONE.

[90] Yang X., Wang X., Ji L., Yi Z., Fu C., Ran J., Hu R., Zhou G. (2015). Overexpression of a *Miscanthus lutarioriparius* NAC gene *MlNAC5* confers enhanced drought and cold tolerance in *Arabidopsis*. Plant Cell Rep..

[91] Liu X., Hong L., Li X.-Y., Yao Y., Hu B., Li L. (2011). Improved drought and salt tolerance in transgenic *Arabidopsis* overexpressing a NAC transcriptional factor from *Arachis hypogaea*. Biosci. Biotechnol. Biochem..

[92] Gao F., Xiong A., Peng R., Jin X., Xu J., Zhu B., Chen J., Yao Q. (2010). *OsNAC52*, a rice NAC transcription factor, potentially responds to ABA and confers drought tolerance in transgenic plants. Plant Cell Tiss. Org. Cult..

[93] Xie L.-N., Chen M., Min D.-H., Feng L., Xu Z.-S., Zhou Y.-B., Xu D.-B., Li L.-C., Ma Y.-Z., Zhang X.-H. (2017). The NAC–like transcription factor *SiNAC110* in foxtail millet (*Setaria italica* L.) confers tolerance to drought and high salt stress through an ABA–independent signaling pathway. J. Integr. Agric..

[94] Qin F., Sakuma Y., Tran L.-S.P., Maruyama K., Kidokoro S., Fujita Y., Fujita M., Umezawa T., Sawano Y., Miyazono K.-I. (2008). *Arabidopsis* DREB2A–interacting proteins function as RING E3 ligases and negatively regulate plant drought stress–responsive gene expression. Plant Cell.

[95] Clough S.J., Bent A.F. (1998). Floral dip: A simplified method for *Agrobacterium*–mediated transformation of *Arabidopsis thaliana*. Plant J..

[96] Tizaoui K., Kchouk M.E. (2012). Genetic approaches for studying transgene inheritance and genetic recombination in three successive generations of transformed tobacco. Genet. Mol. Biol..

[97] Li Y., Varala K., Moose S.P., Hudson M.E. (2012). The inheritance pattern of 24 nt siRNA clusters in *Arabidopsis* hybrids is influenced by proximity to transposable elements. PLoS ONE.

[98] Zhang L., Zhang L., Xia C., Zhao G., Jia J., Kong X. (2016). The novel wheat transcription factor TaNAC47 enhances multiple abiotic stress tolerances in transgenic plants. Front. Plant Sci..

[99] Patterson B.D., MacRae E.A., Ferguson I.B. (1984). Estimation of hydrogen peroxide in plant extracts using titanium(IV). Anal. Biochem..

[100] Bradford M.M. (1976). A rapid and sensitive method for the quantitation of microgram quantities of protein utilizing the principle of protein–dye binding. Anal. Biochem..

[101] Giannopolitis C.N., Ries S.K. (1977). Superoxide dismutases: I. Occurrence in higher plants. Plant Physiol..

[102] Shannon L.M., Kay E., Lew J.Y. (1966). Peroxidase isozymes from horseradish roots. I. Isolation and physical properties. J. Biol. Chem..

[103] Wang C.-J., Yang W., Wang C., Gu C., Niu D.-D., Liu H.-X., Wang Y.-P., Guo J.-H. (2012). Induction of drought tolerance in cucumber plants by a consortium of three plant growth–promoting Rhizobacterium strains. PLoS ONE.

[104] Yang L., Liu Q., Liu Z., Yang H., Wang J., Li X., Yang Y. (2016). *Arabidopsis* C3HC4–ring finger E3 ubiquitin ligase AtAIRP4 positively regulates stress–responsive abscisic acid signaling. J. Integr. Plant Biol..

[105] Livak K.J., Schmittgen T.D. (2001). Analysis of relative gene expression data using real–time quantitative PCR and the 2^−ΔΔCT^ method. Methods.

[106] Huang Y., Sun M.-M., Ye Q., Wu X.-Q., Wu W.-H., Chen Y.-F. (2017). Abscisic acid modulates seed germination via ABA INSENSITIVE5–Mediated PHOSPHATE1. Plant Physiol..

