# Supplementary material for: The Soybean GmNAC019 Transcription Factor Mediates Drought Tolerance in Arabidopsis in an Abscisic Acid-Dependent Manner
Source: Int J Mol Sci. 2019 Dec 31;21(1):286. doi: 10.3390/ijms21010286 (PMC6981368; doi:10.3390/ijms21010286)
Supplement: Supplementary file 1 [file ijms-21-00286-s001.pdf]

Supplementary Table

Table S1. Genes and primers used for RT-qPCR analysis.

No.	Gene	ID	Forward Primer (5'-3')	Reverse Primer (5'-3')	References	E*
1	*CSD1*	At1g08830	AGACCCTGATGACCTCGGAAA	GCCACACACCAGAAGATACAC	[1]	1.8
2	*CAT2*	At4g35090	AACTCCGCCTGCTGTCTG	ATAGGGCATCAATCCATC	[2]	1.9
3	*ACTIN2*	At3g18780	GCACCACCTGAAAGGAAGTACA	CGATTCCTGGACCTGCCTCATC	[3]	1.9
4	*GmNAC019*	Glyma04g38990.1	CGAACGTGTTCAATGTGTCTCT	CCACTCACGCTGGCATTA	[4]	1.8

*As calculated by LinRegPCR software.

References

1. Chen, Y.; Jiang, J.; Song, A.; Chen, S.; Shan, H.; Luo, H.; Gu, C.; Sun, J.; Zhu, L.; Fang, W.; et al. Ambient temperature enhanced freezing tolerance *of Chrysanthemum dichrum CdICE1 Arabidopsis* via miR398. *BMC Biol.* **2013**, *11*, 121.
2. Du, Y.Y.; Wang, P.-C.; Chen, J.; Song, C.-P. Comprehensive functional analysis of the catalase gene family in *Arabidopsis thaliana*. *J. Integr. Plant Biol.* **2008**, *50*, 1318–1326.
3. Yang, L.; Liu, Q.; Liu, Z.; Yang, H.; Wang, J.; Li, X.; Yang, Y. *Arabidopsis* C3HC4–ring finger E3 ubiquitin ligase AtAIRP4 positively regulates stress-responsive abscisic acid signaling. *J. Integr. Plant Biol.* **2016**, *58*, 67–80.
4. Thu, N.B.A.; Hoang, X.L.T.; Doan, H.; Nguyen, T.-H.; Bui, D.; Thao, N.P.; Tran, L.-S.P. Differential expression analysis of a subset of *GmNAC* genes in shoots of two contrasting drought-responsive soybean cultivars DT51 and MTD720 under normal and drought conditions. *Mol. Biol. Rep.* **2014**, *41*, 5563–5569.
